# {5-Meth­oxy-2-[(2-morpholinoethyl)­iminometh­yl]phenolato}(thio­cyanato-κ*N*)­nickel(II)

**DOI:** 10.1107/S160053681000468X

**Published:** 2010-02-13

**Authors:** Lin Liu

**Affiliations:** a College of Chemistry and Biology Engineering, Yichun University, Yichun 336000, People’s Republic of China

## Abstract

In the mononuclear title complex, [Ni(C_14_H_19_N_2_O_3_)(NCS)], the nickel(II) atom is four-coordinated in a square-planar geometry by the O and N atoms of the tridentate Schiff base ligand and by the N atom of a thio­cyanate ligand. The crystal structure is stabilized by inter­molecular C—H⋯S and C—H⋯O hydrogen bonds, forming a three-dimensional network.

## Related literature

For general background to nickel(II) complexes with Schiff bases, see: Campbell & Urbach (1973 ▶); Wallis & Cummings (1974 ▶); Polt *et al.* (2003 ▶); Mukhopadhyay *et al.* (2003 ▶). For related structures, see: Liu (2010 ▶); Montazerozohori *et al.* (2009 ▶); Zhu *et al.* (2004 ▶, 2006 ▶).
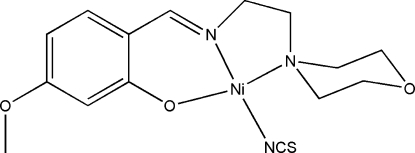


## Experimental

### 

#### Crystal data

[Ni(C_14_H_19_N_2_O_3_)(NCS)]
*M* *_r_* = 380.10
Monoclinic, 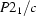

*a* = 12.3983 (18) Å
*b* = 11.8202 (17) Å
*c* = 12.2913 (18) Å
β = 114.756 (2)°
*V* = 1635.8 (4) Å^3^
*Z* = 4
Mo *K*α radiation
μ = 1.33 mm^−1^
*T* = 298 K
0.20 × 0.20 × 0.18 mm

#### Data collection

Bruker SMART CCD area-detector diffractometer
Absorption correction: multi-scan (*SADABS*; Sheldrick, 1996 ▶) *T*_min_ = 0.777, *T*_max_ = 0.796
9279 measured reflections
3554 independent reflections
2729 reflections with *I* > 2σ(*I*)
*R*_int_ = 0.033

#### Refinement

*R*[*F* ^2^ > 2σ(*F* ^2^)] = 0.036
*wR*(*F* ^2^) = 0.090
*S* = 1.03
3554 reflections
209 parameters
H-atom parameters constrained
Δρ_max_ = 0.35 e Å^−3^
Δρ_min_ = −0.38 e Å^−3^

### 

Data collection: *SMART* (Bruker, 1998 ▶); cell refinement: *SAINT* (Bruker, 1998 ▶); data reduction: *SAINT*; program(s) used to solve structure: *SHELXS97* (Sheldrick, 2008 ▶); program(s) used to refine structure: *SHELXL97* (Sheldrick, 2008 ▶); molecular graphics: *SHELXTL* (Sheldrick, 2008 ▶); software used to prepare material for publication: *SHELXTL*.

## Supplementary Material

Crystal structure: contains datablocks global, I. DOI: 10.1107/S160053681000468X/rz2416sup1.cif

Structure factors: contains datablocks I. DOI: 10.1107/S160053681000468X/rz2416Isup2.hkl

Additional supplementary materials:  crystallographic information; 3D view; checkCIF report

## Figures and Tables

**Table 1:** Hydrogen-bond geometry (Å, °)

*D*—H⋯*A*	*D*—H	H⋯*A*	*D*⋯*A*	*D*—H⋯*A*
C3—H3⋯O3^i^	0.93	2.40	3.313 (4)	165
C7—H7⋯O2^ii^	0.93	2.44	3.329 (4)	160
C10—H10*B*⋯S1^iii^	0.97	2.87	3.797 (2)	161
C13—H13*A*⋯O1^iv^	0.97	2.49	3.432 (3)	165

Symmetry codes: (i) 
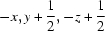
; (ii) 
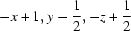
; (iii) 
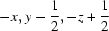
; (iv) 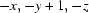.

**Fig. 1.**
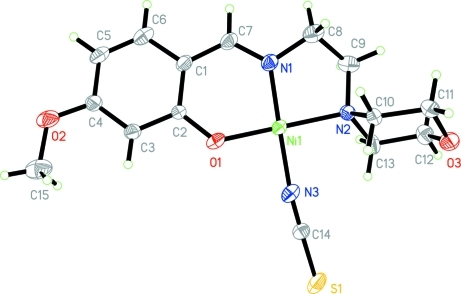
The molecular structure of the title complex, with displacement ellipsoids drawn at the 30% probability level.

## supplementary crystallographic information

### Comment

Nickel(II) complexes with Schiff bases have been extensively studied (Campbell & Urbach, 1973; Wallis & Cummings, 1974; Polt *et al.*, 2003; Mukhopadhyay *et al.*, 2003). Recently, the author reported a nickel(II) complex with the Schiff base 2-[2-(ethylamino)ethyliminomethyl]-5-methoxyphenol (Liu, 2010). In this paper, the crystal structure of the title new nickel(II) complex, with the Schiff base 5-methoxy-2-[(2-morpholin-4-ylethylimino)methyl]phenol, is reported.

The Ni atom in the title complex is four-coordinate by the phenolate O atom, imine N atom, and amine N atom of the Schiff base ligand, and by the N atom of a thiocyanate ligand, forming a square-planar geometry (Fig. 1). The bond lengths and angles involving the metal atom are comparable with those observed in similar complexes (Montazerozohori *et al.*, 2009; Zhu *et al.*, 2004; Zhu *et al.*, 2006). In the crystal structure, the complex molecules are linked into a three-dimensional network by intermolecular C—H···S and C—H···O hydrogen bonds (Table 1).

### Experimental

Equimolar quantities (0.1 mmol) of 4-methoxysalicylaldehyde, *N*-(2-aminoethyl)morpholine, ammonium thiocyanate, and Ni(CH_3_COO)_2_.4H_2_O were mixed and stirred in a methanol solution for 30 min at reflux. After keeping the filtrate in air for a few days, red block crystals suitable for X-ray analysis were formed.

### Refinement

H atoms were placed in calculated positions and constrained to ride on their parent atoms, with C—H distances in the range 0.93–0.97 Å, and with *U*_iso_(H) = 1.2*U*_eq_(C) or 1.5*U*_eq_(C) for methyl H atoms.

### Crystal data

**Table:**

[Ni(C_14_H_19_N_2_O_3_)(NCS)]	*F*(000) = 792
*M**_r_* = 380.10	*D*_x_ = 1.543 Mg m^−^^3^
Monoclinic, *P*2_1_/*c*	Mo *K*α radiation, λ = 0.71073 Å
Hall symbol: -P 2ybc	Cell parameters from 2524 reflections
*a* = 12.3983 (18) Å	θ = 2.5–25.6°
*b* = 11.8202 (17) Å	µ = 1.33 mm^−^^1^
*c* = 12.2913 (18) Å	*T* = 298 K
β = 114.756 (2)°	Block, red
*V* = 1635.8 (4) Å^3^	0.20 × 0.20 × 0.18 mm
*Z* = 4	

### Data collection

**Table:**

Bruker SMART CCD area-detector diffractometer	3554 independent reflections
Radiation source: fine-focus sealed tube	2729 reflections with *I* > 2σ(*I*)
graphite	*R*_int_ = 0.033
ω scan	θ_max_ = 27.0°, θ_min_ = 1.8°
Absorption correction: multi-scan (*SADABS*; Sheldrick, 1996)	*h* = −15→15
*T*_min_ = 0.777, *T*_max_ = 0.796	*k* = −7→15
9279 measured reflections	*l* = −15→15

### Refinement

**Table:**

Refinement on *F*^2^	Primary atom site location: structure-invariant direct methods
Least-squares matrix: full	Secondary atom site location: difference Fourier map
*R*[*F*^2^ > 2σ(*F*^2^)] = 0.036	Hydrogen site location: inferred from neighbouring sites
*wR*(*F*^2^) = 0.090	H-atom parameters constrained
*S* = 1.03	*w* = 1/[σ^2^(*F*_o_^2^) + (0.0433*P*)^2^ + 0.1781*P*] where *P* = (*F*_o_^2^ + 2*F*_c_^2^)/3
3554 reflections	(Δ/σ)_max_ < 0.001
209 parameters	Δρ_max_ = 0.35 e Å^−^^3^
0 restraints	Δρ_min_ = −0.38 e Å^−^^3^

### Special details

**Table:**

Geometry. All esds (except the esd in the dihedral angle between two l.s. planes) are estimated using the full covariance matrix. The cell esds are taken into account individually in the estimation of esds in distances, angles and torsion angles; correlations between esds in cell parameters are only used when they are defined by crystal symmetry. An approximate (isotropic) treatment of cell esds is used for estimating esds involving l.s. planes.
Refinement. Refinement of *F*^2^ against ALL reflections. The weighted *R*-factor wR and goodness of fit *S* are based on *F*^2^, conventional *R*-factors *R* are based on *F*, with *F* set to zero for negative *F*^2^. The threshold expression of *F*^2^ > σ(*F*^2^) is used only for calculating *R*-factors(gt) etc. and is not relevant to the choice of reflections for refinement. *R*-factors based on *F*^2^ are statistically about twice as large as those based on *F*, and *R*- factors based on ALL data will be even larger.

### Fractional atomic coordinates and isotropic or equivalent isotropic displacement parameters (Å^2^)

**Table:**

	*x*	*y*	*z*	*U*_iso_*/*U*_eq_	
Ni1	0.08568 (2)	0.38142 (2)	0.14197 (3)	0.03183 (11)	
O1	0.19256 (14)	0.46933 (15)	0.26251 (14)	0.0392 (4)	
O2	0.53990 (16)	0.69070 (18)	0.46786 (17)	0.0579 (5)	
O3	−0.24289 (16)	0.14271 (16)	−0.05535 (17)	0.0499 (5)	
S1	−0.23786 (6)	0.53989 (8)	0.18241 (7)	0.0594 (2)	
N1	0.19869 (17)	0.32497 (18)	0.09614 (18)	0.0377 (5)	
N2	−0.02732 (16)	0.27875 (16)	0.02024 (16)	0.0334 (4)	
N3	−0.03493 (18)	0.44345 (18)	0.17904 (19)	0.0426 (5)	
C1	0.3595 (2)	0.4495 (2)	0.2126 (2)	0.0393 (6)	
C2	0.3021 (2)	0.4969 (2)	0.2800 (2)	0.0345 (5)	
C3	0.3625 (2)	0.5777 (2)	0.3677 (2)	0.0385 (6)	
H3	0.3264	0.6089	0.4135	0.046*	
C4	0.4760 (2)	0.6113 (2)	0.3864 (2)	0.0435 (6)	
C5	0.5327 (2)	0.5647 (3)	0.3200 (3)	0.0542 (8)	
H5	0.6090	0.5878	0.3335	0.065*	
C6	0.4760 (2)	0.4856 (3)	0.2358 (3)	0.0529 (7)	
H6	0.5143	0.4543	0.1921	0.064*	
C7	0.3061 (2)	0.3638 (2)	0.1276 (2)	0.0428 (6)	
H7	0.3510	0.3321	0.0909	0.051*	
C8	0.1593 (2)	0.2292 (2)	0.0126 (3)	0.0496 (7)	
H8A	0.1724	0.1583	0.0560	0.060*	
H8B	0.2023	0.2274	−0.0376	0.060*	
C9	0.0292 (2)	0.2477 (2)	−0.0620 (2)	0.0472 (7)	
H9A	0.0177	0.3079	−0.1194	0.057*	
H9B	−0.0067	0.1792	−0.1057	0.057*	
C10	−0.0414 (2)	0.1787 (2)	0.0893 (2)	0.0375 (6)	
H10A	−0.0668	0.2055	0.1495	0.045*	
H10B	0.0351	0.1422	0.1306	0.045*	
C11	−0.1300 (2)	0.0925 (2)	0.0110 (2)	0.0457 (6)	
H11A	−0.1010	0.0594	−0.0439	0.055*	
H11B	−0.1381	0.0323	0.0608	0.055*	
C12	−0.2340 (2)	0.2320 (2)	−0.1291 (2)	0.0500 (7)	
H12A	−0.3120	0.2644	−0.1744	0.060*	
H12B	−0.2052	0.2021	−0.1855	0.060*	
C13	−0.1501 (2)	0.3231 (2)	−0.0532 (2)	0.0450 (7)	
H13A	−0.1456	0.3830	−0.1051	0.054*	
H13B	−0.1817	0.3555	−0.0001	0.054*	
C14	−0.1188 (2)	0.4842 (2)	0.1815 (2)	0.0378 (6)	
C15	0.4866 (3)	0.7462 (3)	0.5363 (3)	0.0716 (10)	
H15A	0.4169	0.7862	0.4833	0.107*	
H15B	0.5421	0.7988	0.5907	0.107*	
H15C	0.4648	0.6911	0.5808	0.107*	

### Atomic displacement parameters (Å^2^)

**Table:**

	*U*^11^	*U*^22^	*U*^33^	*U*^12^	*U*^13^	*U*^23^
Ni1	0.02692 (17)	0.03345 (18)	0.03502 (18)	−0.00070 (13)	0.01288 (13)	−0.00172 (14)
O1	0.0278 (8)	0.0510 (11)	0.0406 (9)	−0.0070 (8)	0.0160 (7)	−0.0087 (8)
O2	0.0449 (11)	0.0704 (14)	0.0562 (12)	−0.0265 (10)	0.0191 (10)	−0.0103 (11)
O3	0.0369 (10)	0.0518 (11)	0.0553 (11)	−0.0091 (9)	0.0137 (9)	0.0012 (9)
S1	0.0378 (4)	0.0820 (6)	0.0614 (5)	0.0141 (4)	0.0236 (4)	−0.0038 (4)
N1	0.0352 (11)	0.0367 (11)	0.0437 (12)	0.0009 (10)	0.0191 (10)	−0.0043 (10)
N2	0.0334 (10)	0.0337 (11)	0.0327 (10)	−0.0013 (9)	0.0134 (9)	0.0018 (9)
N3	0.0310 (11)	0.0456 (13)	0.0508 (13)	−0.0024 (10)	0.0167 (10)	−0.0102 (11)
C1	0.0289 (12)	0.0427 (15)	0.0472 (15)	0.0005 (11)	0.0167 (11)	−0.0002 (12)
C2	0.0273 (12)	0.0379 (13)	0.0379 (13)	0.0005 (11)	0.0134 (10)	0.0079 (11)
C3	0.0337 (13)	0.0445 (14)	0.0371 (13)	−0.0046 (12)	0.0146 (11)	0.0019 (12)
C4	0.0334 (13)	0.0514 (16)	0.0389 (14)	−0.0097 (13)	0.0083 (11)	0.0047 (13)
C5	0.0308 (13)	0.066 (2)	0.0664 (19)	−0.0087 (14)	0.0213 (14)	−0.0015 (16)
C6	0.0359 (14)	0.0619 (18)	0.0669 (18)	−0.0026 (14)	0.0274 (14)	−0.0084 (16)
C7	0.0354 (13)	0.0467 (16)	0.0521 (16)	0.0054 (12)	0.0240 (13)	−0.0019 (13)
C8	0.0513 (16)	0.0465 (16)	0.0613 (17)	−0.0045 (14)	0.0337 (14)	−0.0153 (14)
C9	0.0575 (17)	0.0485 (16)	0.0409 (14)	−0.0134 (14)	0.0260 (13)	−0.0090 (13)
C10	0.0371 (13)	0.0365 (13)	0.0369 (13)	0.0031 (11)	0.0134 (11)	0.0073 (11)
C11	0.0504 (16)	0.0358 (14)	0.0523 (16)	−0.0048 (13)	0.0229 (14)	0.0030 (12)
C12	0.0429 (15)	0.0519 (17)	0.0408 (15)	−0.0032 (14)	0.0034 (12)	0.0020 (13)
C13	0.0412 (14)	0.0381 (14)	0.0402 (14)	0.0031 (12)	0.0018 (12)	0.0064 (12)
C14	0.0351 (13)	0.0406 (14)	0.0373 (13)	−0.0026 (12)	0.0148 (11)	−0.0042 (11)
C15	0.066 (2)	0.086 (2)	0.065 (2)	−0.037 (2)	0.0303 (18)	−0.0246 (19)

### Geometric parameters (Å, °)

**Table:**

Ni1—N1	1.840 (2)	C5—C6	1.353 (4)
Ni1—O1	1.8402 (16)	C5—H5	0.9300
Ni1—N3	1.885 (2)	C6—H6	0.9300
Ni1—N2	1.9796 (19)	C7—H7	0.9300
O1—C2	1.323 (3)	C8—C9	1.500 (4)
O2—C4	1.359 (3)	C8—H8A	0.9700
O2—C15	1.429 (4)	C8—H8B	0.9700
O3—C11	1.421 (3)	C9—H9A	0.9700
O3—C12	1.425 (3)	C9—H9B	0.9700
S1—C14	1.621 (3)	C10—C11	1.512 (3)
N1—C7	1.304 (3)	C10—H10A	0.9700
N1—C8	1.468 (3)	C10—H10B	0.9700
N2—C9	1.497 (3)	C11—H11A	0.9700
N2—C13	1.502 (3)	C11—H11B	0.9700
N2—C10	1.508 (3)	C12—C13	1.517 (3)
N3—C14	1.158 (3)	C12—H12A	0.9700
C1—C7	1.406 (3)	C12—H12B	0.9700
C1—C2	1.414 (3)	C13—H13A	0.9700
C1—C6	1.415 (3)	C13—H13B	0.9700
C2—C3	1.400 (3)	C15—H15A	0.9600
C3—C4	1.385 (3)	C15—H15B	0.9600
C3—H3	0.9300	C15—H15C	0.9600
C4—C5	1.394 (4)		
			
N1—Ni1—O1	93.86 (8)	C9—C8—H8A	110.6
N1—Ni1—N3	176.31 (9)	N1—C8—H8B	110.6
O1—Ni1—N3	87.85 (8)	C9—C8—H8B	110.6
N1—Ni1—N2	86.27 (8)	H8A—C8—H8B	108.7
O1—Ni1—N2	176.19 (8)	N2—C9—C8	108.1 (2)
N3—Ni1—N2	92.24 (8)	N2—C9—H9A	110.1
C2—O1—Ni1	127.84 (15)	C8—C9—H9A	110.1
C4—O2—C15	118.7 (2)	N2—C9—H9B	110.1
C11—O3—C12	110.8 (2)	C8—C9—H9B	110.1
C7—N1—C8	118.6 (2)	H9A—C9—H9B	108.4
C7—N1—Ni1	126.50 (18)	N2—C10—C11	113.25 (19)
C8—N1—Ni1	114.88 (15)	N2—C10—H10A	108.9
C9—N2—C13	108.85 (19)	C11—C10—H10A	108.9
C9—N2—C10	112.65 (19)	N2—C10—H10B	108.9
C13—N2—C10	106.55 (18)	C11—C10—H10B	108.9
C9—N2—Ni1	106.12 (14)	H10A—C10—H10B	107.7
C13—N2—Ni1	117.30 (15)	O3—C11—C10	111.2 (2)
C10—N2—Ni1	105.51 (13)	O3—C11—H11A	109.4
C14—N3—Ni1	168.7 (2)	C10—C11—H11A	109.4
C7—C1—C2	121.6 (2)	O3—C11—H11B	109.4
C7—C1—C6	119.4 (2)	C10—C11—H11B	109.4
C2—C1—C6	119.0 (2)	H11A—C11—H11B	108.0
O1—C2—C3	118.4 (2)	O3—C12—C13	110.5 (2)
O1—C2—C1	122.8 (2)	O3—C12—H12A	109.5
C3—C2—C1	118.8 (2)	C13—C12—H12A	109.5
C4—C3—C2	120.2 (2)	O3—C12—H12B	109.5
C4—C3—H3	119.9	C13—C12—H12B	109.5
C2—C3—H3	119.9	H12A—C12—H12B	108.1
O2—C4—C3	123.9 (3)	N2—C13—C12	112.5 (2)
O2—C4—C5	115.0 (2)	N2—C13—H13A	109.1
C3—C4—C5	121.0 (3)	C12—C13—H13A	109.1
C6—C5—C4	119.5 (2)	N2—C13—H13B	109.1
C6—C5—H5	120.2	C12—C13—H13B	109.1
C4—C5—H5	120.2	H13A—C13—H13B	107.8
C5—C6—C1	121.4 (3)	N3—C14—S1	178.8 (2)
C5—C6—H6	119.3	O2—C15—H15A	109.5
C1—C6—H6	119.3	O2—C15—H15B	109.5
N1—C7—C1	125.4 (2)	H15A—C15—H15B	109.5
N1—C7—H7	117.3	O2—C15—H15C	109.5
C1—C7—H7	117.3	H15A—C15—H15C	109.5
N1—C8—C9	105.9 (2)	H15B—C15—H15C	109.5
N1—C8—H8A	110.6		

### Hydrogen-bond geometry (Å, °)

**Table:**

*D*—H···*A*	*D*—H	H···*A*	*D*···*A*	*D*—H···*A*
C3—H3···O3^i^	0.93	2.40	3.313 (4)	165
C7—H7···O2^ii^	0.93	2.44	3.329 (4)	160
C10—H10B···S1^iii^	0.97	2.87	3.797 (2)	161
C13—H13A···O1^iv^	0.97	2.49	3.432 (3)	165

Symmetry codes: (i) −*x*, *y*+1/2, −*z*+1/2; (ii) −*x*+1, *y*−1/2, −*z*+1/2; (iii) −*x*, *y*−1/2, −*z*+1/2; (iv) −*x*, −*y*+1, −*z*.
